# Multisensory Origin of the Subjective First-Person Perspective: Visual, Tactile, and Vestibular Mechanisms

**DOI:** 10.1371/journal.pone.0061751

**Published:** 2013-04-22

**Authors:** Christian Pfeiffer, Christophe Lopez, Valentin Schmutz, Julio Angel Duenas, Roberto Martuzzi, Olaf Blanke

**Affiliations:** 1 Center for Neuroprosthetics, School of Life Sciences, Ecole Polytechnique Fédérale de Lausanne, Lausanne, Switzerland; 2 Laboratory of Cognitive Neuroscience, Brain Mind Institute, School of Life Sciences, Ecole Polytechnique Fédérale de Lausanne, Lausanne, Switzerland; 3 Laboratoire de Neurosciences Intégratives et Adaptatives, UMR 7260, Centre National de la Recherche Scientifique and Aix-Marseille Université, Marseille, France; 4 Rehabilitation Engineering Lab, Institute for Robotics and Intelligent Systems, Eidgenössische Technische Hochschule Zürich, Zürich, Switzerland; 5 Department of Neurology, University Hospital Geneva, Geneva, Switzerland; Royal Holloway, University of London, United Kingdom

## Abstract

In three experiments we investigated the effects of visuo-tactile and visuo-vestibular conflict about the direction of gravity on three aspects of bodily self-consciousness: self-identification, self-location, and the experienced direction of the first-person perspective. Robotic visuo-tactile stimulation was administered to 78 participants in three experiments. Additionally, we presented participants with a virtual body as seen from an elevated and downward-directed perspective while they were lying supine and were therefore receiving vestibular and postural cues about an upward-directed perspective. Under these conditions, we studied the effects of different degrees of visuo-vestibular conflict, repeated measurements during illusion induction, and the relationship to a classical measure of visuo-vestibular integration. Extending earlier findings on experimentally induced changes in bodily self-consciousness, we show that self-identification does not depend on the experienced direction of the first-person perspective, whereas self-location does. Changes in bodily self-consciousness depend on visual gravitational signals. Individual differences in the experienced direction of first-person perspective correlated with individual differences in visuo-vestibular integration. Our data reveal important contributions of visuo-vestibular gravitational cues to bodily self-consciousness. In particular we show that the experienced direction of the first-person perspective depends on the integration of visual, vestibular, and tactile signals, as well as on individual differences in idiosyncratic visuo-vestibular strategies.

## Introduction

Recent research investigated how the processing of bodily signals modulates bodily self-consciousness and in particular *self-location* (i.e. the experience of where ‘I’ am in space) and *self-identification* with the body (i.e. the experience of identifying and owning a body) [1], [2], [3], [4], [5]. In these studies participants were presented with conflicting multisensory stimuli (such as visual, tactile, and proprioceptive signals) about the location and appearance of a body part (e.g. rubber hand illusion: [2]; enfacement illusion: [6], [7]) or their entire body (e.g. full-body illusion: [8], [9]).

Concerning the full-body illusion several paradigms have been used to investigate self-identification and self-location and their underlying brain mechanisms [10], [11]. Changes in self-identification and self-location towards a virtual body have been induced in participants who were exposed to visuo-tactile mismatch between their own body and a filmed or virtual body [1], [3], [4], [9], [12] and have been associated with physiological changes [4], [12], changes in visuo-tactile integration [1], [13], and decreases in pain perception [14].

More recently, the effects of different visuo-spatial viewpoints on self-identification with a virtual body have been tested [15], [16], [17]. These studies investigated self-identification with a virtual body that was seen from a first- or third-person viewpoint and revealed stronger self-identification for first- than third-person viewpoints. Other studies have identified distinct behavioural and neural mechanisms when participants employed first-person as compared to a third-person viewpoints in perspective taking paradigms (i.e. [18], [19], [20]). Although these studies are important for cognitive mechanisms of perspective taking and highlight the effects of different visuo-spatial viewpoints on the strength of self-identification, they do not allow to induce changes in more subjective aspects of *first-person perspective*, that is the experience from where ‘I’ perceive the world [10], [11].

**Figure 1:**
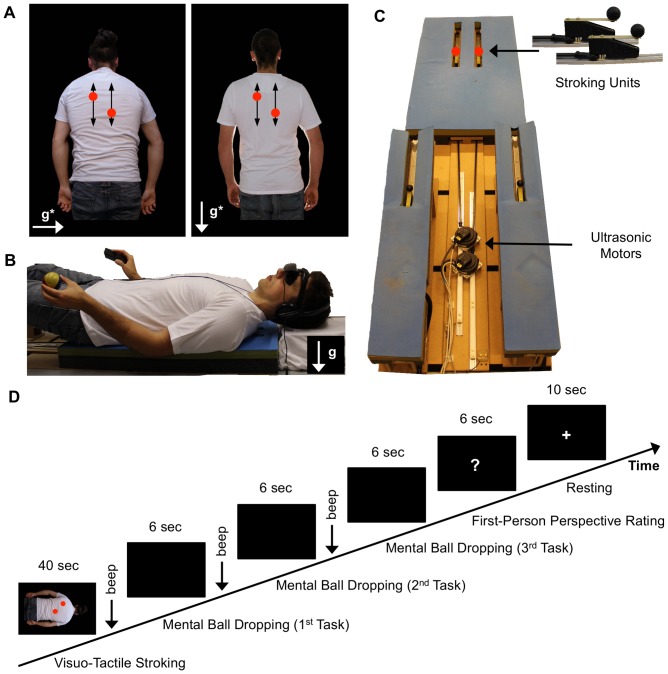
Experimental stimuli, setup, and procedure. (A) Visual stimuli showing a virtual body in prone posture from an elevated downward perspective used during the strong Visuo-Vestibular Conflict condition (left panel) and the same body standing used during the weak Visuo-Vestibular Conflict condition (right panel). Visual implied direction of gravity in each panel is indicated by a white arrow and ‘g*’ label. Visual stroking was presented by red dots (trajectory indicated by black arrows). (B) Participant lying supine, equipped with a ball to facilitate mental imagery during Mental Ball Dropping task, a button response device, and a head-mounted display. Direction of veridical vertical is indicated by a white arrow and ‘g’ label. (C) Robotic device used for tactile stimulation of the participants’ back. Stroking units (in red color) were actuated by ultrasonic motors. (D) Sequence of events in an experimental trial.

This was achieved in a recent study where changes in the experienced direction of the first-person perspective were induced in the absence of any overt visual changes that were present in all previous works on the first-person perspective. The participants in the study by Ionta et al. [21] were lying supine on a robotic device with their head oriented upwards and their arms outstretched next to their body. They wore a head-mounted display (HMD) and saw the back of a virtual body as if seen from an elevated and downward looking perspective (Fig. 1A left panel and Fig. 1B). Participants were thus exposed to strong visuo-vestibular conflict. All participants received robot-controlled visuo-tactile stimulation. Yet, despite identical visuo-tactile stimulation, there were individual differences in the direction of the experienced first-person perspective. Half of the participants experienced looking upwards to the virtual body (‘Up-group’), whereas the other half experienced looking downwards to the virtual body (‘Down-group’). These individual differences in the experienced direction of the first-person perspective were associated with congruent patterns of self-location.

Ionta et al. [21] argued that these individual differences in the experienced direction of the first-person perspective were related to individual differences in multisensory integration of visual and vestibular signals related to gravity. Thus, participants in that study viewed a visual image on the HMD that contained a conflict between the visual gravitational cues of the seen body and the gravitational acceleration coded by the participant’s vestibular and somatosensory receptors [22], [23], [24], [25]. This may have caused differences in the experienced direction of the first-person perspective, with participants from the Up-group relying more strongly on vestibular cues (indicating gravitational acceleration directed towards the participants’ body) than on visual gravitational cues from the seen virtual body (indicating gravitational acceleration away from the participants’ body), whereas participants from the Down-group showed the opposite pattern. However, individual differences in visuo-vestibular integration and their relevance for first-person perspective and other aspects of bodily self-consciousness have not yet been tested.

In the present series of experiments, we used a robotic full-body illusion paradigm and studied the multisensory mechanisms of self-identification, self-location and, in particular, of the experienced direction of the first-person perspective. In Experiment 1, we investigated whether different degrees of visuo-vestibular conflict have a distinct impact on self-identification, self-location, and the experienced direction of the first-person perspective. In Experiment 2, we investigated whether individual differences in the experienced direction of the first-person perspective that we observed in Experiment 1 for strong visuo-vestibular conflicts could also be quantified in repeated judgments of first-person perspective. In Experiment 3, we investigated whether individual differences in the experienced direction of the first-person perspective during the full-body illusion correlated with idiosyncratic differences in visuo-vestibular integration as quantified in a classical task of visuo-vestibular integration (i.e. visual vertical judgments).

### Ethics Statement

All experimental protocols were approved by the local ethics committee–La Commission d’Ethique de la Recherche Clinique de la Faculté et de Medicine de l’Université de Lausanne–and each experiments was conducted in line with the Declaration of Helsinki. The person on the photographs of Figure 1 has given written informed consent, as outlined in the PLOS consent form, to publication of their photograph. Participants gave written informed consent to participate in the experiment before inclusion in the experiment.

## Experiment 1

In Experiment 1, we investigated whether and how different levels of visuo-vestibular conflict during the full-body illusion, and thus additional visuo-tactile stimulation, influence bodily self-consciousness, in particular the experienced direction of the first-person perspective. For this, we used a modified version of the robotic device, that was used by Ionta et al. [21], and virtual reality technology to precisely control visuo-tactile stroking. We manipulated visuo-vestibular conflict by presenting participants with visual cues about the direction of gravity, which did not match the direction of veridical vertical (Fig. 1A–B). We hypothesized, first, that visuo-tactile stroking modulates self-identification (i.e. [1], [4], [9], [12]) and that visuo-tactile stroking, together with visuo-vestibular conflict, would modulate self-location and the experienced direction of the first-person perspective. Following Ionta et al. [21] we used first-person perspective ratings to divide the participants sample into two subgroups that differed in terms of their predominantly experienced direction of the first-person perspective (Up- versus Down-group participants, see below). Based on findings by Ionta et al. [21], we hypothesized, second, that self-location but not self-identification would reflect individual differences between first-person perspective groups.

### Methods

#### Participants

Twenty-six students from the Ecole Polytechnique Fédérale de Lausanne participated participated (12 female; mean age: 21 years, range: 18–28 years). All participants were right-handed, had normal or corrected-to-normal vision, and reported no history of neurological or psychiatric impairment. Each participant was debriefed about the experimental purpose and received 30 CHF after the experiment.

#### Experimental setup

The experiment was conducted in complete darkness. A custom-made robotic device was installed on a table at 90 cm above the floor. The robotic device had 200 cm×90 cm×10 cm dimensions (for a detailed description see [26]). Figure 1B–C illustrates the experimental setup with a participant lying on the robotic device.

The robotic device stroked the back of the participant with two stroking units. A stroking unit consisted of an ultrasonic motor (Shinsei, USR60-E3N, Japan, http://www.shinsei-motor.com) delivering rotatory motion, a carbon stick that translated rotatory in linear motion, and a sliding unit with a plastic sphere mounted that touched the back of the participant. The stroking units stroked the left and right upper back of participants. Soft foam covered the robotic device to allow participants to comfortably lie during prolonged periods. The foam included gaps permitting the plastic spheres to directly touch the back of the participant. Participants wore a cotton T-shirt in order to reduce frictions between the plastic sphere and their back.

Visual stimuli were presented to participants on a head-mounted display (HMD, Virtual Realities, Virtual Viewer 3D, www.vrealities.com/virtualviewer3d.html) with a resolution of 800×600 pixels, representing about 35° of visual angle. Headphones presented white noise to participants to mask acoustic cues from robotic stroking. In-house software (ExpyVR, http://lnco.epfl.ch/expyvr) was used for visual stimulus presentation, real-time synchronization of visual stroking with robotic stroking, and for recording responses of the participant. Participants gave their responses with their right hand on a serial keypad (Targus Numeric Keypad AKP10US, www.targus.com).

#### Visual and tactile stimuli

Participants were presented with conflicting visuo-tactile stroking to induce the full-body illusion. ‘*Visual stroking’* consisted of projecting two red dots on the back of a virtual body seen in the HMD. The red dots moved along pre-defined stroking paths (illustrated by black arrows in Fig. 1A). *‘Tactile stroking’* consisted of moving two plastic spheres along the back of a participant lying on the robotic device (Fig. 1C).

The sequences of visual stroking (seen on the HMD) and tactile stroking (felt on the participant’s back) were either synchronous or asynchronous. Four stroking profiles were created before the experiment. Each profile consisted of a random sequence of positions in 0–20 cm distance range, 2–12 cm/s velocity range, and 40 s duration. The stroking profiles varied randomly in length, speed, direction, and inter-stroke-intervals (0–1.5 s), thus when simultanously executed they were incongruent. During the experiment, either two times the same profile or two incongruent profiles were randomly assigned to a stroking unit (touching the back of the participant) and the corresponding red dot (on the HMD), which resulted in visuo-tactile synchronous or asynchronous stroking.

Participants saw on the HMD a virtual body filmed from its back at 2 m distance, who wore a white T-shirt and blue jeans. All visual information around the virtual body was removed and replaced by black color in order to exclude visual cues about absolute distance to the environment. Either a male or female virtual body was shown to match the participant’s gender. Male and female virtual body size was matched, as well as overall luminance in the images. We were careful to match the limb configuration of participants, who were lying on the robotic device, to the limb configuration of the virtual body, seen in the HMD. Participants’ arms were positioned next to their trunk on the soft foam and their limbs were outstretched on the robotic device.

In addition to visuo-tactile stroking we manipulated visuo-vestibular conflicts about the direction of linear gravitational acceleration. We presented in the HMD images that showed a virtual body (seen from the back) in different postures with respect to visual gravity. Visual gravity cues were gravitational pull on hair, clothes, and the posture of the shoulders of the virtual body [27], [28]. In addition, we chose a distribution of light on the front and back of the virtual body that was congruent with a light following the direction of visual gravity.

The first image (Fig. 1A, right panel) showed a virtual body in prone posture on which linear gravitational acceleration acted along an axis through the virtual body’s back and chest. This image gave the impression of looking downwards at the virtual body. The second image (Fig. 1A, left panel) showed the same virtual body in standing posture on which linear gravitational acceleration acted along a vertical axis from the virtual body’s head and feet. This image gave the impression of looking in front at the virtual body. The two images (i.e. looking downwards, looking in front) were respectively in strong and weak visual-vestibular conflict with the participant posture lying on the back on the robotic device and looking upwards (Fig. 1B). For the first image (strong conflict), the conflict was of 180° and for the second image (weak conflict) it was of 90°.

#### Experimental procedures and data collection

Each participant completed 32 trials in 4 experimental runs of 8 trials each. For each experimental run the 8 trials were from the same condition, but the stroking profiles were randomly selected for each trial. Fig. 1D illustrates the organization of each trial. Each trial began with the presentation of visual stroking on the virtual body in the HMD while tactile stroking was applied on the back of the participant for 40 s. After that, participants were shown a blank screen for a fixed inter-stimulus interval of 1 s. An acoustic beep was presented for 200 ms that instructed participants to perform the Mental Ball Dropping task within 6 s.

The Mental Ball Dropping task (adapted from [3], [21]) was used to measure self-location. Before the experiment proper, participants performed a training session with at least 20 trials to be familiarized with the experimental procedures and the materials. Participants were asked to imagine dropping a ball from their hand to the floor (Fig. 1B). First, they pressed a button with their right index finger when they imaged dropping a ball from their hand, which was at the level of their body lying supine. Participants held the button depressed during the imagined time of ball dropping and released the button at the moment they imagined the ball hit the floor. The duration of button press (response time, RT) was shown to be a sensitive estimate of the participant’s height, or self-location, above the floor [3]. Participants executed three Mental Ball Dropping tasks successively, then a white fixation cross was presented for 20 s, indicating a pause before the next experimental trial.

After having completed 8 trials of an experimental run participants answered a short version of the full-body illusion questionnaire (adapted from [3], [21], [29]. Questions were presented separately on the HMD along with a visual analogue scale, i.e. a continuous visual scale from left to right with either two or 11 levels, on which participants indicated their response. The questions measured (1) self-identification, by rating their agreement with the statement *“It felt as if the body I saw was me”* using a 11-point visual analogue scale ranging from 1 ( = weak feeling) to 11 ( = strong feeling); (2) illusory touch, by rating their agreement with the statement *“I had the feeling as if the touch I felt was located where I saw the stroking”* using a 11-point visual analogue scale ranging from 1 ( = weak feeling) to 11 ( = strong feeling); (3) and the experienced direction of the first-person perspective, by answering the question *“Did you have the impression as if you were looking upwards/downwards at a body above/below you?”* with a forced-choice categorical response format labeled 0 ( = “*upwards*”) and 1 ( = “*downwards*”).

#### Data analysis

Individual answers to question 3 regarding the experienced direction of first-person perspective were used to assign participants to two groups. Following the methods of Ionta et al. [21], who reported individual differences in first-person perspective and self-location, participants were assigned to the Up-group, if less than 2 out of the total 4 ratings were downward direction of the experienced first-person perspective (N = 15). Participants were assigned to the Down-group, if at least 2 out of 4 ratings were downward direction of the first-person perspective (N = 9). The Group (Up-group, Down-group) was used as a between-participants factor for subsequent statistical analyses.

Questionnaire scores for self-identification (question 1), illusory touch (question 2), and first-person perspective (question 3) were analyzed using separate 2×2×2 mixed model ANOVAs with one between-participants factor Group (levels: Up-group, Down-group) and two within-participants factors Visuo-Vestibular Conflict (levels: strong, weak) and Stroking (levels: synchronous, asynchronous).

For the Mental Ball Dropping task (i.e. self-location measure), we excluded trials that contained no response and trials with response times shorter than 200 ms or longer than 4 s. We excluded the data from two participants from further analysis because more than 10% of their trials had to be excluded. For the remaining 24 participants, we removed response times that exceeded 2 standard deviations of the grand average. We calculated then, for each participant, trial-wise averages across three repetitions of the Mental Ball Dropping task and used this data to calculate condition-wise averages for the four experimental conditions. Mean response times were analyzed with a 2×2×2 mixed model ANOVA with one between-participant factor Group (levels: Up-group, Down-group) and two within-participant factors Visuo-Vestibular Conflict (levels: strong, weak) and Stroking (levels: synchronous, asynchronous).

Post-hoc comparisons were performed with an a priori alpha level of.05. As post-hoc comparisons were conducted only the basis of significant interactions in ANOVAs, there was no correction for multiple comparisons.

### Results

#### Questionnaire scores

**Figure 2:**
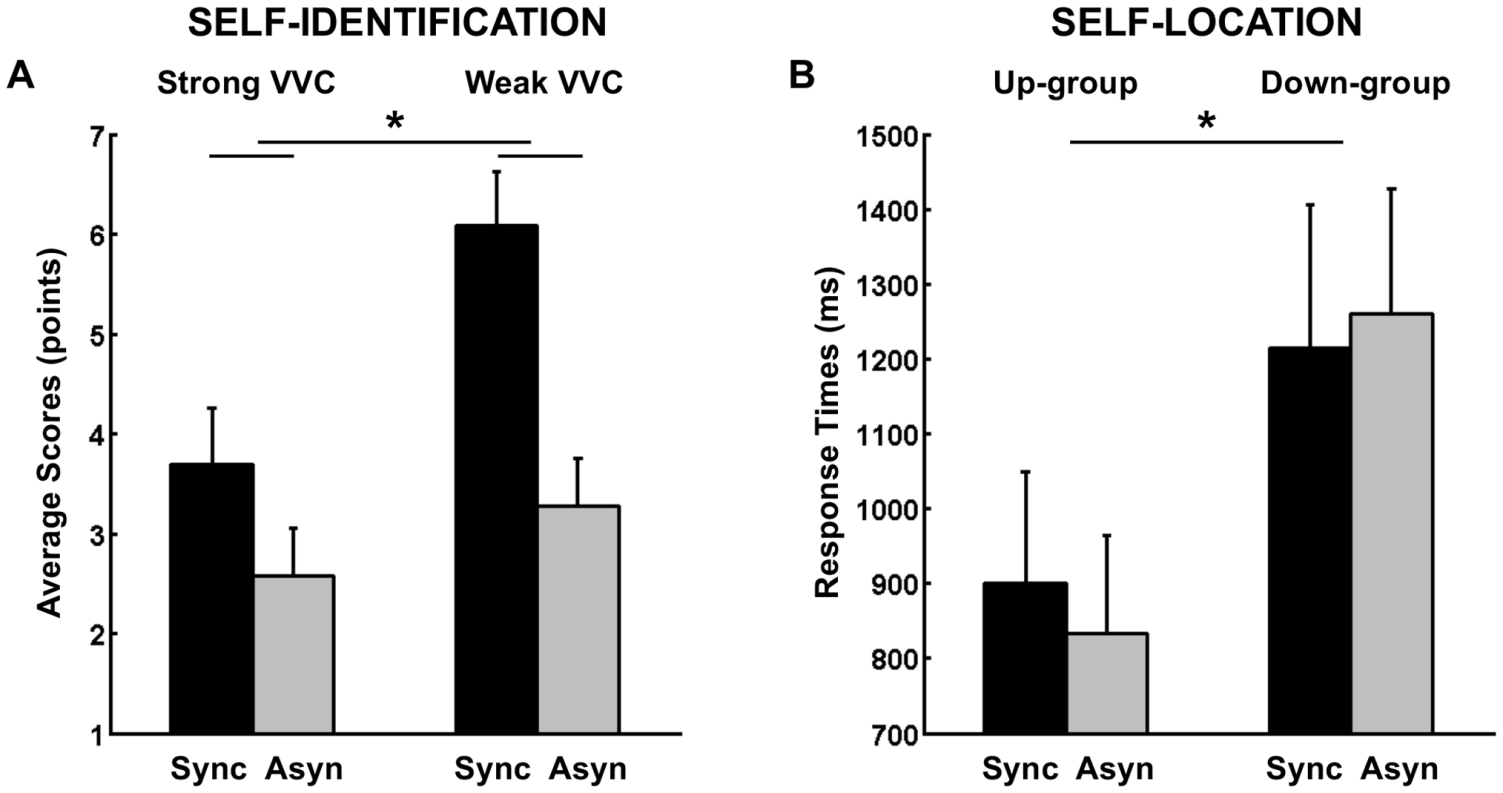
Results of Experiment 1. (A) Average self-identification ratings for the synchronous (Sync) and asynchronous (Asyn) visual-tactile stroking and for the strong and weak visual-vestibular conflict (VVC). (B) Self-location results showing average response times in the Mental Ball Dropping task. Error bars indicate standard errors of the mean.

Statistical analysis of self-identification ratings (question 1) revealed a main effect of Stroking (F(1, 22) = 24.06, p<.001, η^2^ = .52). Participants rated on average 5.9 (SE = .5) points for synchronous Stroking and 2.9 (SE = .4) points for asynchronous Stroking. This main effect reflects that synchronous visuo-tactile Stroking increased self-identification with a virtual body and shows that we induced the full-body illusion with a novel robotic device. In addition we found a main effect of Visuo-Vestibular Conflict on self-identification (F(1, 22) = 16.25, p = .001, η^2^ = .43). Participants rated 3.1 (SE = .5) points for strong Visuo-Vestibular Conflict and 4.7 (SE = .4) points for weak Visuo-Vestibular Conflict, suggesting that our manipulation of visuo-vestibular conflict had an influence on self-identification and that a strong visuo-vestibular conflict decreases self-identification with the virtual body. Furthermore, we found a significant interaction of Visuo-Vestibular Conflict×Stroking regarding self-identification (F(1,22) = 9.35, p = .006, η^2^ = .30, Fig. 2A). Self-identification with the virtual body decreased during strong Visuo-Vestibular Conflict in the synchronous conditions (post-hoc paired t-test, t(23) = −4.8, p<.001), but not in the asynchronous control conditions. There were no main effect and interactions involving the between-participant factor Group (all F values <1), reflecting that individual differences in the direction of first-person perspective had no influence on self-identification.

Statistical analysis of illusory touch ratings (question 2) showed a main effect of Stroking (F(1,22) = 152.69, p<.001, η^2^ = .87). Participants rated illusory touch on average with 9.0 (SE = .2) points for synchronous Stroking and 2.6 (SE = .5) points for asynchronous Stroking. This main effect shows that synchronous Stroking induced stronger illusory touch sensation. No other main effects and interactions were significant (F <1).

#### Response times

Statistical analysis of response times of the Mental Ball Dropping task revealed an interaction of Stroking × Group, F(1,22) = 4.85, p = .038, η^2^ = .18 (Fig. 2B). Up-group participants’ response times were on average 901 (SE = 149) ms for synchronous Stroking and 834 (SE = 130) ms for asynchronous stroking. By contrast, for the Down-group participants, the pattern of response times was reversed and averaged 1214 (SE = 192) ms for synchronous Stroking and 1260 (SE = 167) ms for asynchronous Stroking. Post-hoc tests revealed a marginally significant difference between response times of the two Groups for asynchronous Stroking (independent samples t-test, t(22) = 2.0, p = .056). These results corroborate data by Ionta et al. [21] and reveal that those participants who experience mostly an upward direction of the first-person perspective showed longer response times in the synchronous versus asynchronous Stroking condition (indicating a drift in self-location towards the seen virtual body). This was different in participants experiencing mostly a downward direction of the first-person perspective who showed the opposite drift (i.e. a decrease in response times). In both groups, we observed a drift in self-location towards the seen virtual body. No other effects were significant.

#### First-person perspective ratings

Following the methods of Ionta et al. [21], we used first-person perspective ratings to divide the total sample of participants into Up-group (N = 15) and Down-group (N = 9) (see Data analysis section).

Statistical analysis of first-person perspective ratings (question 3) only revealed a main effect of Group, F(1,10) = 94.3, p<.001, η^2^ = .81, which is a direct consequence of our method using question 3 rating to assign the datasets into two Groups. Up-group participants rated on average.08 (SE = .04) points and Down-group participants rated.75 (SE = .05) points. These scores reflect average frequency of participants rating *“downwards”* direction of first-person perspective (i.e. because we assigned the value 0 for an *“upwards”* rating and the value 1 for a *“downwards”* rating). The main effect reflects lower frequency of *“downwards”* rating for the Up-group and a higher frequency of *“downwards”* rating for the Down-group. No other main effect and no interaction reached statistical significance (F <1), reflecting that Stroking and Visuo-Vestibular Conflict did not influence first-person perspective.

### Discussion

In Experiment 1, we investigated how different degrees of Visuo-Vestibular Conflict modulate self-identification, the experienced direction of first-person perspective, and self-location during the full-body illusion. To this end, we used a novel robotic device [26] to administer visuo-tactile stimulation and manipulated the degree of Visuo-Vestibular Conflict and the synchrony of Stroking.

Regarding self-identification (question 1), we found, as expected, an increase in self-identification with the virtual body for synchronous stroking, supporting several earlier video and virtual reality studies applying visuo-tactile stroking manually [1], [4], [6], [9], [12], [30]. We also confirmed that self-identification does not depend on the experienced direction of the first-person perspective [21]. A new finding was that self-identification additionally depended on the degree of Visuo-Vestibular Conflict, with strong conflict decreasing self-identification, an effect found only for the illusion condition (during synchronous visuo-tactile stimulation). These data show that under conditions of illusory self-identification with the virtual body strong Visuo-Vestibular Conflict decreases illusory self-identification, suggesting that the visuo-vestibular compatibility between the participant’s body posture and position and those of the virtual body interfere with self-identification (see General Discussion).

Concerning the first-person perspective, we asked participants to rate their experienced direction once at the end of each experimental condition. Similarly to Ionta et al. [21], we found individual differences in Up- and Down-group participants. Using a slightly modified robotic platform, different experimental conditions, and a different participants sample we also observed for Up-group (respectively, Down-group) participants that response times increased (decreased) during the synchronous versus asynchronous Stroking condition, indicating a more elevated (lower) self-location in the illusion condition. These self-location data corroborate the presence of individual differences in first-person perspective and demonstrate a directional congruence between the experienced direction of the first-person perspective and the direction of the drift in self-location. However, these subjective ratings did not depend on the tested visuo-vestibular conflict or on visuo-tactile stroking. Therefore, they did not support our hypothesis that visuo-vestibular conflict, as manipulated here, is of relevance for the experienced direction of the first-person perspective.

In conclusion, Experiment 1 revealed that self-identification depends on visuo-vestibular and visuo-tactile mechanisms, whereas self-location and first-person perspective were only modulated by visuo-tactile stimulation. We confirmed the presence of individual differences in self-location and first-person perspective and the dependence of self-location on the experienced direction of the first-person perspective.

## Experiment 2

In Experiment 1, we measured the direction of the first-person perspective once at the end of multiple repeated trials for the same condition. Using this procedure we may have not been able to detect more subtle changes in first-person perspective. Whereas in Experiment 1 trial order was randomized at the level of experimental runs (i.e. all trials within an experimental run were from the same experimental condition), in Experiment 2 trial order was randomized trial-by-trial. Participants were presented with the virtual body in strong visuo-vestibular conflict to be consistent with the study by Ionta et al. [21], and we collected the experienced direction of the first-person perspective after each experimental trial. In addition, we measured self-identification and self-location in a control condition where no body was shown (as in Ionta et al., [21]).

### Methods

#### Participants

Twenty-three students participated (11 female; mean age: 22 years, range: 18–30 years). All participants were right-handed, had normal or corrected-to-normal vision, and reported no history of neurological or psychiatric impairment. Each participant was debriefed about the experimental purpose and received 30 CHF after the experiment.

#### Experimental setup and stimuli

We used an identical experimental setup and the same visuo-tactile stroking stimuli as in Experiment 1. Self-identification, self-location, and the experienced direction of the first-person perspective were tested by presenting in a HMD a virtual body in strong Visuo-Vestibular Conflict (i.e. body condition) or a control condition in which the stroking was shown on a black background (i.e. no-body condition). In contrast to Experiment 1, participants judged their experienced direction of their first-person perspective repeatedly during the full-body illusion.

#### Experimental design and procedures

The full-body illusion was tested in 4 experimental conditions: 2 Object conditions (levels: body, no-body)×2 Stroking conditions (levels: synchronous, asynchronous), which were presented in a pseudo-randomized order. Participants completed 8 trials for each of the 4 experimental conditions. Each trial began with visuo-tactile Stroking for 40 s. Immediately after, all visual stimuli were removed from the display, and after 1 s, an acoustic beep was presented for 200 ms. Participants executed a single Mental Ball Dropping task within 6 s (identical procedure as for Experiment 1). After the Mental Ball Dropping task, they judged the direction of their first-person perspective. In the HMD the question *“Orientation?”* was presented in white color along with a two-choice response scale showing *“upwards”* and *“downwards”*. Participants were instructed to judge after each trial the direction of the first-person perspective experienced during the preceding stroking period. They gave their judgment within 6 s by pressing either a button with their right index finger to indicate an experienced upward direction of the first-person perspective, or by pressing a button with their middle finger to indicate an experienced downward direction of the first-person perspective. A white fixation cross was presented on the HMD for 20 s, indicating a pause before the next trial. In contrast with Experiment 1, participants executed the Mental Ball Dropping task only once and gave a first-person perspective judgment at the end of each experimental trial. In this way, we obtained a measure of self-location and first-person perspective for each experimental trial. After having completed the experiment, participants answered a short-version of the full-body illusion questionnaire separately for synchronous and asynchronous stroking (see Experiment 1).

#### Data analysis

Individual answers to question 3 regarding the experienced direction of first-person perspective (collected once at the end of the experiment) were used to assign participants to two groups (see Experiment 1 for details). We considered participants as Up-group participants when they experienced an upward direction of first-person perspective for both synchronous and asynchronous stroking (Up-group, N = 12). Down-group participants were those who experienced a downward direction of first-person perspective for synchronous and/or asynchronous stroking (Down-group, N = 11). The rationale for this procedure was to balance group size by lowering the threshold for classification. In Experiment 2, the downward direction of the first-person perspective was less frequently reported than upward direction.

Scores for self-identification (question 1) and illusory touch (question 2) were analyzed with separate 2×2 mixed model ANOVAs with one between-participant factor Group (levels: Up-group, Down-group) and one within-participant factor Stroking (levels: synchronous, asynchronous). No self-identification ratings were collected for the no-body condition.

Response times for the Mental Ball Dropping task (i.e. self-location measure) were analyzed as in Experiment 1. Condition-wise average response times for each participant were analyzed using a 2×2×2 mixed model ANOVA with the between-participant factor Group (levels: Up-group, Down-group) and two within-participant factors Object (levels: body, no-body) and Stroking (levels: synchronous, asynchronous).

Judgments of the direction of the first-person perspective given after each trial were analyzed after excluding trials where participant did not give a judgment within 6 s (<10%). We coded *“upwards”* responses as 0 and *“downwards”* responses as 1. Individual frequencies of *“downwards”* rating were calculated for each condition (i.e. sum of the values across the repetitions of each condition divided by the total number of valid judgments per condition). We thus obtained, for each participant and each condition, a frequency value of *“downwards”* rating ranging from 0 (i.e. never judged *“downwards”*) to 1 (i.e. always judged *“downwards”*). These frequencies were analyzed with a 2×2×2 mixed model ANOVA with the between-participant factor Group (levels: Up-group, Down-group) and two within-participant factors Object (levels: body, no-body) and Stroking (levels: synchronous, asynchronous).

### Results

#### Questionnaire scores

Statistical analysis of self-identification (question 1) revealed a main effect of Stroking (F(1, 21) = 11.9, p = .002, η^2^ = .36), reflecting higher self-identification for synchronous stroking (mean ± SE: 4.3±.5) than for asynchronous stroking (2.8±.4). There was no difference between the two Groups and no interaction (all F values <1). As for Experiment 1, visuo-tactile synchrony influenced self-identification with a virtual body and individual differences in the experienced direction of first-person perspective did not modulate self-identification.

Statistical analysis of illusory touch (question 2) showed a main effect of Stroking (F(1, 21) = 35.0, p<.001, η^2^ = .63). Illusory touch was higher for synchronous stroking (6.2±.4) than for asynchronous stroking (4.0±.5). There was no difference between the Groups and no significant interaction (all F values <1).

#### Response times

**Figure 3:**
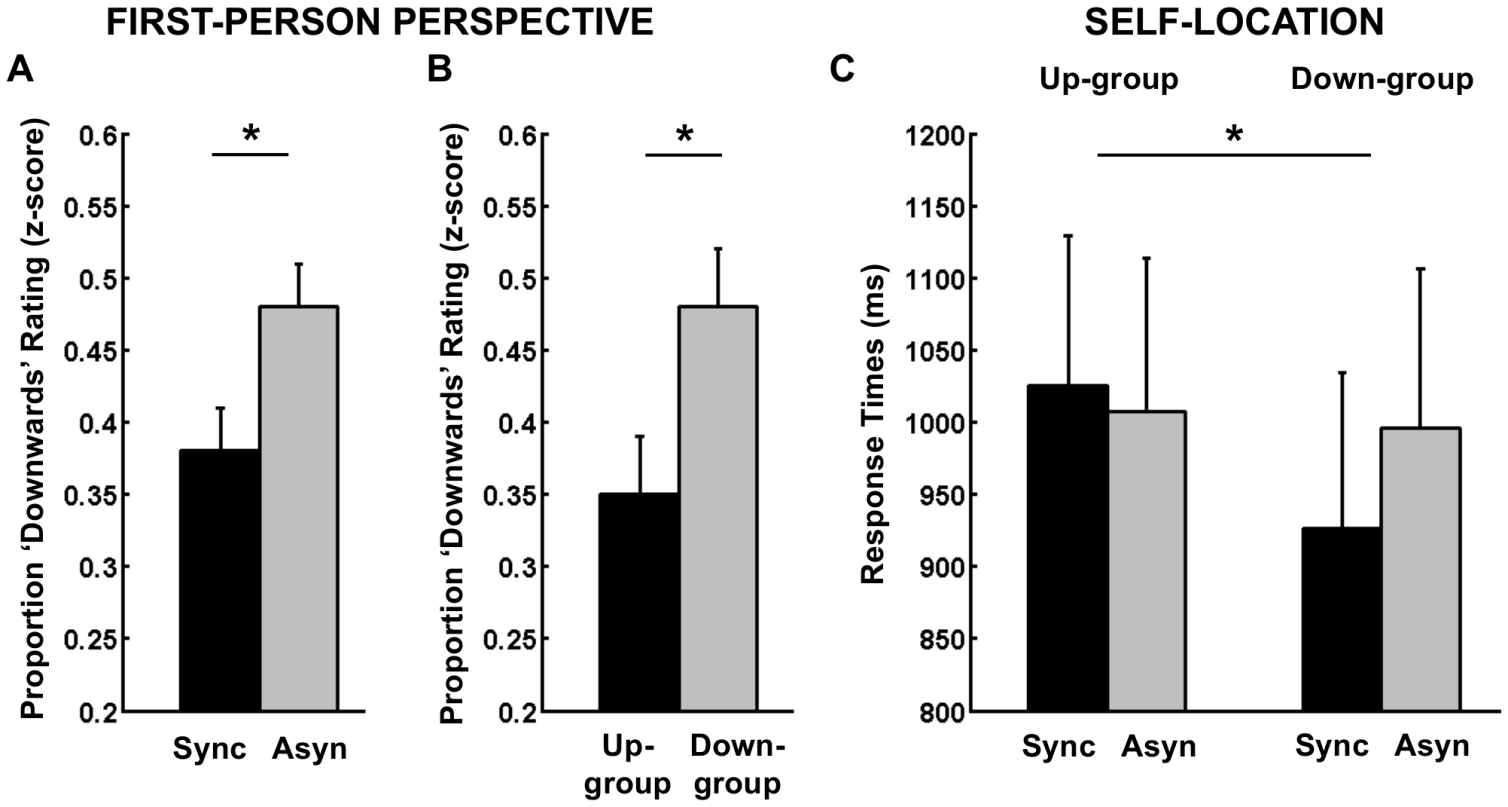
Results of Experiment 2. Frequency of *“downwards”* ratings for the experienced direction of the first-person perspective showing the main effect of Stroking (A) and Group (B). (C) Self-location results showing average response times in the Mental Ball Dropping task. Error bars indicate standard error of the mean.

Statistical analysis of response times of the Mental Ball Dropping task revealed an interaction between Stroking and Group (F(1,21) = 6.87, p = .016, η^2^ = .25, Fig. 3C). For Up-group participants response times were on average 1025 (SE = 104) ms for synchronous Stroking and 1007 (SE = 107) ms for asynchronous Stroking (paired-sample t-test, p>.1), whereas for Down-group participants response times were 926 (SE = 108) ms in the synchronous and 996 (SE = 110) ms in the asynchronous Stroking condition (paired sample t-test, t(10) = −2.4, p = .04). Thus, we confirmed that the pattern of self-location (as measured through response times) is congruent with individual differences in the experienced direction of the first-person perspective. Up-group participants showed an upward drift in self-location (a slight increase in response times during the illusion) congruent with the upward direction of the first-person perspective, By contrast, Down-group participants show a downward drift in self-location (a decrease in response time during the illusion) congruent with the experienced downward direction of the first-person perspective.

The analysis also revealed a marginally significant effect of Object (F(1,21) = 4.1, p = .057, η^2^ = .16) with longer response times for the body condition (M = 992, SE = 76 ms) when compared to the no-body control condition (M = 974, SE = 76 ms). This suggests that independently of visuo-tactile Stroking, self-location tended to be more elevated if a body in strong Visuo-Vestibular Conflict was presented as compared to a no-body control condition. There was no significant effect of Group and no interaction (all F values <1).

#### First-person perspective ratings

Statistical analysis of first-person perspective ratings collected after each trial revealed a significant main effect of Stroking (F(1,21) = 6.23, p = .021, η^2^ = .23, Fig. 3A). The frequency of *“downwards”* ratings was higher during asynchronous (M = .48, SE = .03) than synchronous Stroking (M = .38, SE = .03). Furthermore, the analysis showed a main effect of Group (F(1,21) = 5.88, p = .024, η^2^ = .22, Fig. 3B), with an average *“downwards”* rating frequency of.35 (SE = .04) for the Up-group and.48 (SE = .04) for the Down-group. There was no significant main effect of Object and no significant interactions (all F values <1). These results confirm the consistency between final ratings of first-person perspective in the full-body illusion questionnaire (i.e. on which grouping was based) and trial-wise ratings for first-person perspective during the experiment. However, they also show that trial-wise ratings fluctuated for all participants and that a downward direction of the first-person perspective was more likely associated with asynchronous visuo-tactile stimulation.

### Discussion

The data on self-identification and self-location corroborate those of Experiment 1 and previous work in a different participant sample. We found that self-identification and illusory touch were higher in the synchronous Stroking condition and that the degree of self-identification was not related to individual differences in the experienced direction of first-person perspective. Self-location was found to be Stroking- and first-person perspective-dependent and we again observed a relative elevation in self-location towards the seen virtual body for Up-group participants and a relative lowering in self-location for Down-group participants. In Experiment 2, the order of the trials was randomized to control for habituation and training effects. This differed from previous studies (Experiment 1, [21]), further underlining the consistency of these changes in self-location and self-identification.

Self-location results are consistent across three experiments–the study by Ionta et al. [21] and the present Experiments 1 and 2. The asynchronous Stroking condition induced significantly higher self-location for Down-group participants when compared to Up-group participants. Subjective first-person perspective was most frequently rated *“downwards”* in the asynchronous Stroking condition (Experiment 2), although self-identification was low ([21]; Experiments 1–2). Furthermore, the body condition, where a virtual body was presented in strong visuo-vestibular conflict, was associated with higher self-location than the no-body condition. Together these results suggest that asynchronous stroking and the presentation of a virtual body in strong visuo-vestibular conflict induced a response pattern congruent with the visually-implied direction of gravity: high self-location, downwards direction of the subjective first-person perspective, and low self-identification.

In Experiment 2, participants indicated their experienced direction of the first-person perspective after each 40-second period of visuo-tactile stimulation. Analysis of trial-wise ratings confirmed those ratings of the first-person perspective collected at the end of Experiment 2. Although this reveals consistency of ratings given in the full-body illusion at different time points, the first-person perspective data from Experiment 2 also showed that participants that were classified as Up- or Down-group may also have experienced quite frequently a direction of the first-person perspective that was 180° inverted with respect to their most frequently experienced perspective. This may be compared to effects found in bistable perception in which identical physical stimuli evoke two perceptual states that alternate spontaneously [31], [32], [33].

Moreover, our analysis revealed an influence of visuo-tactile Stroking on the frequency of downward direction of the first-person perspective. We found a higher frequency of downward direction of the first-person perspective during asynchronous Stroking conditions as compared to synchronous Stroking conditions. Ionta et al. [21] found that self-location depended on both first-person perspective and visuo-tactile stroking. In this study, Down-group participants showed higher self-location in the asynchronous than in the synchronous stroking condition, reflecting that self-location decreased or drifted towards the seen virtual body (in line with the downwards direction of subjective first-person perspective). Thus, during asynchronous stroking, an association of a downwards direction of the subjective first-person perspective with a high level of self-location was found [21]. The same participants reported in the same asynchronous condition floating sensations, sensations of being elevated and of touching the ceiling, compatible with more elevated self-location. Thus, first-person perspective ratings, self-location measures, and spontaneous verbal reports were strongly related during asynchronous visuo-tactile stroking. In Experiment 2, we found an association between asynchronous visuo-tactile Stroking and a downward direction of the experienced first-person perspective, not only for Down-group participants, but for all participants.

In conclusion, Experiment 2 shows that within participants the synchrony of visuo-tactile Stroking affected the first-person perspective, resulting in the highest frequency of downward direction of the first-person perspective for asynchronous Stroking. Furthermore, self-location was elevated above a no-body baseline condition level when a virtual body was shown in strong visuo-vestibular conflict from an elevated viewpoint. Finally, we confirmed the results of individual differences in self-location and first-person perspective from Experiment 1 and by Ionta et al. [21]. Together, these results suggest that both visuo-tactile integration and individual differences can affect the experienced direction of the first-person perspective.

## Experiment 3

In Experiment 3, we investigated whether individual differences in first-person perspective are associated with idiosyncratic strategies for solving visual-vestibular conflicts. Individual differences and strategies for processing visual-vestibular mismatch have traditionally been approached by tasks requiring visual vertical judgments. Visual vertical judgments require the integration of vestibular signals (informing about the direction of gravity), somatosensory signals (informing about the position of the body segments) and visual signals (informing about the orientation of the visual environment) [34]. Typically, participants are required to align a visual line with their internal representation of the vertical [35]. The influence of visual signals on vertical perception has been investigated by manipulating the orientation of the visual background relative to the veridical vertical to induce visual-vestibular conflicts. In the widely used rod and frame test, participants judge the orientation of a mobile rod that is embedded in a tilted square frame [35]. The perceived visual vertical is typically deviated in the direction of the frame tilt. However, the amplitude of this deviation is strongly variable across subjects and depends on the degree to which participants rely on visual references. Two groups of participants have usually been dissociated [23], [24], [35], [36], [37]. Visual Field-Dependent (FD) participants present strong deviations of the perceived vertical in the direction of the frame tilt, indicating that they rely strongly on visual signals. By contrast, visual Field-Independent (FI) participants present smaller deviations of the perceived vertical, indicating that they rely more on vestibular and somatosensory signals. It has been argued that visual field dependence-independence is a stable trait, which shows a high robustness throughout life [38], [39]. As the rod and frame test is a well-established way to measure individual differences in visuo-vestibular integration, the present experiment directly investigates how visual field dependence-independence relates to the experienced direction of the first-person perspective. We hypothesize that FD participants, in contrast with FI participants, are more likely to experience a direction of the first-person perspective that is congruent with that visually shown in the HMD. Data from Experiment 1 suggest that these participants should be more prone to rely on the visually conflicting gravitational information when exposed to strong Visuo-Vestibular Conflict. Thus, in the present case, FD participants observing a body lying in a prone position should experience more frequently a downward direction of the first-person perspective.

### Methods

#### Participants

Twenty-nine students participated (11 female; mean age: 23 years, range: 18–30 years). All participants were right-handed, had normal or corrected-to-normal vision, and reported no history of neurological or psychiatric impairment. Each participant was debriefed about the experimental purpose and received 40 CHF after the experiment.

#### Methods for the rod and frame test

Participants were comfortably seated in front of a computer screen (Philips 150S6FS, TFT, 1024×768 resolution, 60 Hz refresh rate) at 60 cm eye-to-screen distance. The screen was covered with a black circular frame in order to restrict the visual field to a circular area (36 cm in diameter, subtending 34° of the visual field) and to exclude any vertical and horizontal references from the visual surrounding (for similar methods see [40]). A chinrest was used to maintain the participants’ line-of-gaze aligned with the center of the screen. Participants wore custom-made goggles to occlude any visual cue surrounding the circular-shaped screen.

A grey dotted line (18 cm long, subtending 17° of the visual field) was presented on the screen. This line was surrounded by a square frame (22×22 cm, subtending 29° of the visual field), which was either vertical or tilted by 20° in the clockwise or counterclockwise direction. This amplitude of the frame tilt has been shown to evoke large deviations of the perceived visual vertical towards the frame tilt [24], [34], [41]. Participants performed visual vertical judgments by pressing a left or right response button to rotate the line in a clockwise or counterclockwise direction until they judged the line vertically oriented. They were instructed to ignore the surrounding frame and to perform accurate and un-speeded judgments. The initial position in which the line was shown was either clockwise (6 trials) or counterclockwise (6 trials) at pseudo-random offset of ±12°, ±6°, and ±3° from veridical vertical. We used the same frame orientation for six consecutive trials before another frame orientation was presented. Each frame orientation was presented twice, and a total of twelve measurements were obtained per condition. For each participant, we calculated the average subjective visual vertical for each frame orientation (20° counterclockwise, 20° clockwise, vertical frame). Subjective visual vertical was analyzed with repeated-measures ANOVA with the Frame orientation as a within-participants factor (levels: clockwise, counterclockwise, and vertical frame).

#### Methods for the full-body illusion

After having completed the rod and frame test, participants were tested with the full-body illusion paradigm. The procedures were identical to that of Experiment 2, except for one aspect. In order to validate the robustness of the response times during the Mental Ball Dropping task, participants performed this task during and after the stroking.

We used a 2 Object (levels: body, no-body control) ×2 Stroking (levels: synchronous, asynchronous) design. We measured self-location by recording response times in the Mental Ball Dropping task and asked participants to indicate their experienced direction of first-person perspective after each experimental trial (online first-person perspective judgment) and after each experimental block (final first-person perspective judgment). After the experiment, participants filled in a questionnaire about the full-body illusion separately for the synchronous and asynchronous Stroking conditions. Self-identification was not rated for the no-body control condition.

#### Experimental procedures

Each experimental trial began with the presentation of visuo-tactile stroking for 40 s. In contrast to Experiments 1 and 2, participants performed the Mental Ball Dropping task twice during the stroking and twice after the stroking period. We modified the timing of the Mental Ball Dropping task to investigate the possibility to use the Mental Ball Dropping task as an online measure during stroking. An acoustic beep was presented for 200 ms, cueing participants to perform the Mental Ball Dropping task within 6 s, and response times were recorded as the duration of button press. After the stroking, all visual stimuli were removed from the display and the stroking stopped. After the last Mental Ball Dropping task, participants indicated their experienced direction of first-person perspective by a button press. The phrase “*Orientation?*” was presented in the HMD together with three response categories (category 1: “*As if I was looking up at a body above me*”; category 2: “*As if I was looking in front at a standing body*”; category 3: “*As if I was looking down at a body below me*”). Participants indicated their judgments by button press with the right index (for *“upwards”*), middle (for *“front”*), or ring finger (for *“downwards”*). Immediately after, a fixation cross was presented for 10 s, indicating a resting period.

After the experiment, participants gave a final rating of first-person perspective, considering the experiment as a whole, and indicated their most frequently experienced direction of first-person perspective in a forced-choice two-response format (category 1: “*As if I was looking up at a body above me*”; category 2: “*As if I was looking down at a body below me*”). Participants answered the full-body illusion questionnaire separately for the synchronous and the asynchronous stroking condition (11 items, visual presentation of the questions together with a 11-point visual analogue scale, adapted from [3]).

#### Data analysis

As in Experiments 1 and 2, participants were asked for a final rating of their overall experienced direction of first-person perspective. We used this rating to classify participants into Up-group and Down-group. Each item of the full-body illusion questionnaire was analyzed separately using a 2×2 mixed model ANOVA with a between-participants factor Group (levels: Up-group, Down-group) and a within-participant factor Stroking (levels: synchronous, asynchronous).

Response times for the Mental Ball Dropping task were averaged after excluding trials (less than 10%) with responses shorter than 200 ms and longer than 4 s as well as response times that exceeded 2 standard deviations of the grand average. For each trial, we calculated averages of 4 Mental Ball Dropping tasks. These data were analyzed using a mixed model ANOVA with the between-participants factor Group (levels: Up-group, Down-group) and two within-participant factors: Object (levels: body, no-body control), and Stroking (levels: synchronous, asynchronous).

Analysis of trial-wise ratings of first-person perspective-direction included calculation of frequency scores for “downwards” ratings by summing all non-“upwards” ratings (i.e. “downwards” and “front”) per condition and dividing this value by the total number of trials per condition. Because both the “front” and “downwards” response categories were similar in that they indicated deviation from the participants’ physical body orientation (looking “upwards”), we decided to collapse the “front” and “downwards” judgments into a single score reflecting deviation from “upwards”. This resulted in a comparable ratio of Up-group versus Down-group participants as in the previous experiments where no “front” category was used. Thus, frequency scores ranged from 0 (i.e. never “downwards” and never “front”) to 1 (i.e. always “downwards” or “front”). Individual frequency scores for “downwards” first-person perspective were subjected to a mixed model ANOVA with the between-participant factor Group (levels: Up-group, Down-group) and the within-participant factors Object (levels: body, no-body control) and Stroking (levels: synchronous, asynchronous).

#### Field dependence-independence classification and analysis

We analyzed the relationship between field dependence-independence and the experienced direction of the first-person perspective in two ways. First, across all participants we conducted a linear correlation analysis between continuous values for subjective visual vertical bias (average across left and right frame tilt condition) and the frequency of downward first-person perspective (average across experimental conditions).

**Figure 4:**
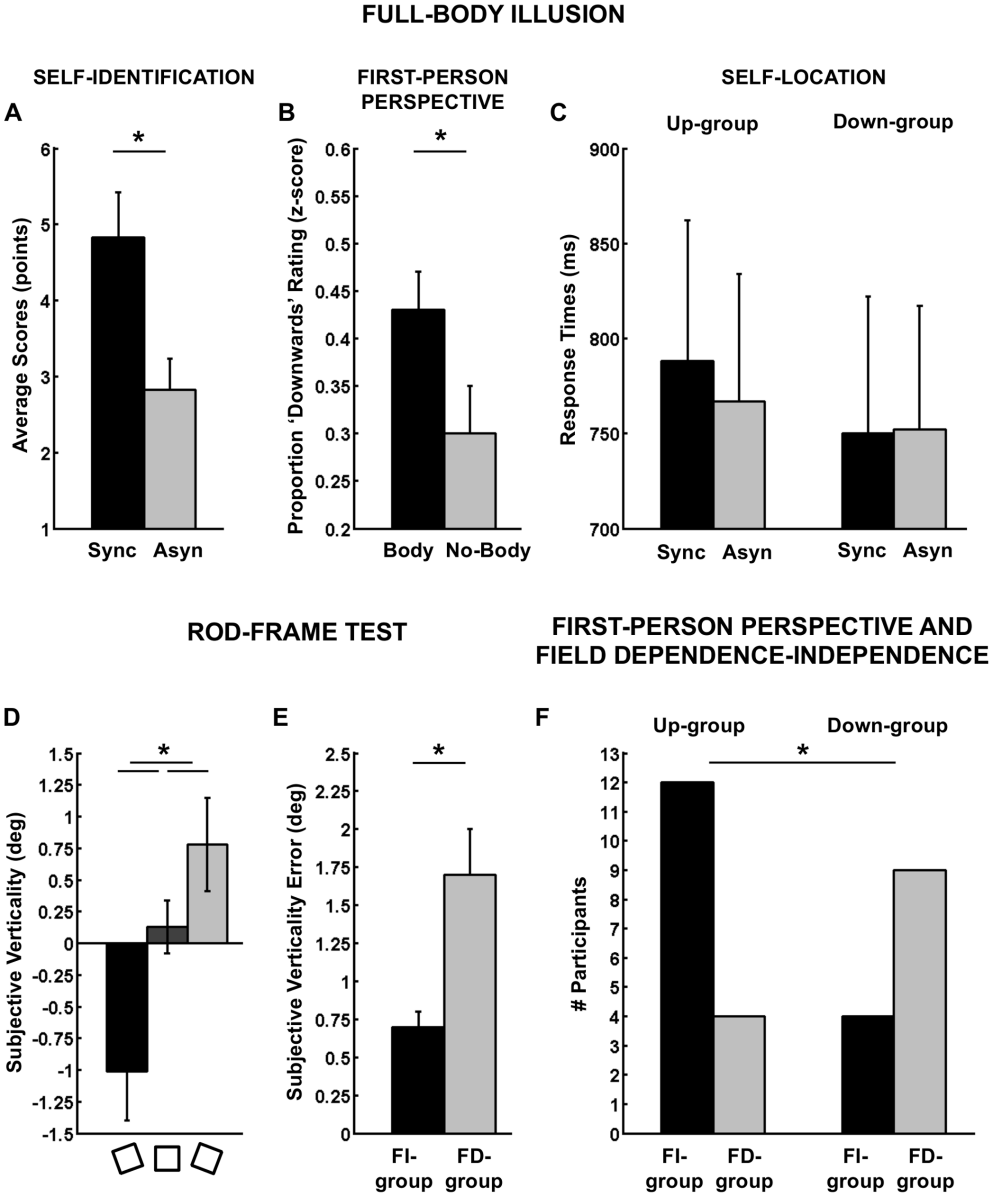
Results of Experiment 3. (A) Main effect of synchrony of visual-tactile Stroking on self-identification. (B) Average frequency of *“downwards”* rating for the experienced direction of the first-person perspective for the virtual body and no-body control condition. (C) Self-location results showing a similar pattern as Experiment 1 and 2. (D) Average subjective verticality rating for different frame orientations. Negative (positive) values denote counterclockwise (clockwise) deviations of the subjective verticality. (E) Average subjective verticality error in field independent (FI-group) and field dependent (FD-group) participants. Error bars denote standard errors of mean. (F) Association between Field dependence-independence and direction of the first-person perspective.

Second, comparing subgroups of participants we performed a binominal correlation analysis (see below) on classification-based labels for field dependence-independence (FI-group, FD-group) and individual difference in first-person perspective (Up-group, Down-group). Data processing involved calculating baseline-corrected averages of subjective visual vertical for each participant by subtracting the perceived vertical measured with the vertical frame to the perceived vertical for the clockwise and counterclockwise frame orientations (see [24] for similar approach). We used an ascending hierarchical classification, i.e. a standard procedure for processing rod and frame test data, to classify participants into two groups of visual field dependent (FD) and field independent (FI) participants (see [24], [42] for similar methods). Ascending hierarchical classification was performed on these data with SPSS 13.0 (IBM corporation, New York, US). The clustering method took into account individual average subjective verticality ratings for left-, and right-frame conditions. The method evaluated similarities between individual ratings of different participants by calculating Euclidean distance between participants. Based on Euclidean distances, the hierarchical clustering algorithm grouped participants into clusters using the Ward’s aggregation method. Ward’s aggregation linked pairs of participants, who were close, into binary clusters forming a hierarchical tree. Finally, separating the hierarchical tree at the maximum of dissimilarity provided two distinct clusters of participants with low (cluster 1, FI-group) or high (cluster 2, FD-group) deviations of perceived vertical induced by the tilted frame (Fig. 4E).

In order to investigate the relationship between labels for visual field dependence-independence and labels for first-person perspective-direction for each participant, we used the phi coefficient as a binominal non-parametric test of correlation [43]. The phi coefficient indicates whether two binominal categorical variables correlate, and in which direction they are associated.

### Results

#### Rod and frame test

The ANOVA revealed a significant main effect of the frame orientation (F(2,27) = 12.0, p<.001, η^2^ = .47, Fig. 4D) with a counterclockwise bias for the frame tilted counterclockwise (M = −1.01°, SE = .39°), a clockwise bias for the frame tilted clockwise (M = .78°, SE = .38°), and no bias for the vertical frame (M = .02°, SE = .21°). The analysis of the visual field dependence by an ascending hierarchical classification revealed a group of 13 FD participants (subjective vertical: M = 1.70°, SE = .30°) that presented significantly larger deviations of the visual vertical than a group of 16 FI participants (subjective vertical: M = .70°, SE = .10) when the frame was tilted by 20° (Fig. 4E).

#### Field dependence-independence correlates with first-person perspective

Linear correlation analysis between continuous data for subjective visual vertical bias and experienced direction of the first-person perspective showed no significant correlation (R = .107, p = .578), suggesting that across the entire participant sample there was no linear relationship between these measures.

Binominal correlation analysis between field dependence-independence and individual differences of the first-person perspective correlated significantly (N = 29, Phi coefficient = -.442, p = .017). Fig. 4F shows that there were proportionally more FI participants in the Up-group (12 out of 16) than in the Down-group (4 out of 13). Conversely, there were proportionally more FD participants in the Down-group (9 out of 13) than in the Up-group (4 out of 16). This result confirms our hypothesis that FD participants rely more on the visual information about the direction of gravity that was contained in the videos depicting a body lying in a prone position. Furthermore, this results shows a relationship between field dependence-independence and first-person persepective on the level of individual differences.

#### Self-identification and self-location

The ANOVA showed a significant effect of stroking for self-identification (question 1). Self-identification was higher during synchronous (M = 4.9 points, SE = .6 points) than asynchronous stroking (M = 2.9 points, SE = .4 points) (F(1,28) = 12.3, p = .002, η^2^ = .31, Fig. 4A). Similarly, illusory touch (question 2) was higher for the synchronous (M = 8.0 points, SE = .4 points) as compared to the asynchronous stroking condition (M = 4.3 points, SE = .5 points) (F(1,27) = 40.4, p<.001, effect size = .60). There were no group differences (i.e. between Up- and Down-group of first-person perspective) in all questionnaire items.

Although, the pattern of Mental Ball Dropping response times was similar to those obtained in Experiment 1 and 2 (Fig. 4C), statistical analysis of the response times revealed no significant main effect and interaction. Inspection of the data shows that Up-group participants showed longer response times in the synchronous (M = 850 ms, SE = 65 ms) versus asynchronous Stroking condition (M = 828 ms, SE = 59 ms), whereas Down-group participants did not show the expected changes in response times (synchronous: M = 666 ms, SE = 72 ms; asynchronous: M = 670 ms, SE = 65 ms).

Regarding online ratings of the first-person perspective, the ANOVA revealed a significant main effect of Object (F(1,27) = 8.8, p = .006, η^2^ = .25), with higher frequency of *“downwards”* ratings in the body condition (M = .43, SE = .04) as compared to the no-body condition (M = .30, SE = .05) (Fig. 4B). These results show that *“downwards”* ratings were more frequent in the condition in which a body was shown (i.e. in strong visuo-vestibular conflict) as compared to a no-body control condition, where no visual cues about the direction of vertical were provided. In addition, the statistical analysis revealed a main effect of Group with a lower frequency of *“downwards”* ratings for the Up-group (M = .14, SE = .05) as compared to the Down-group (M = .59, SE = .06) (F(1,27) = 5.7, p<.001, η^2^ = .55). This result confirms the consistency between online and final ratings of first-person perspective and is also consistent with data from Experiment 2. There were no other main effects or interactions.

### Discussion

Results of the rod and frame test showed that oriented visual references resulted in a predicted bias of visual vertical judgment. Experiment 3 thus replicates with a 2-dimensional computer-adaptation of the rod and frame test earlier findings obtained with the classical 3-dimensional rod and frame test [35], [44]. As noted previously, biases of visual vertical judgments that are measured with a 2-dimentional rod and frame test are weaker, but nonetheless significant (review in [44]). In the present experiment, we classified participants into two groups of FD and FI participants [23], [24], [36] that differed in term of the perceptual bias evoked by a tilted frame. This result reveals individual differences in solving visual-vestibular conflict during the rod and frame test. These differences, also referred to as perceptual styles or sensory strategies, have been related to idiosyncratic selection of spatial frames of reference for spatial perception and orientation [45]. According to this view, we predicted that FD participants will rely mostly on an allocentric (i.e. visual) frame of reference, whereas FI participants will rely mainly on an egocentric (i.e. body-centered) frame of reference. The correlation between the visual field dependence-independence and the experienced direction of first-person perspective is discussed in the General Discussion.

As in previous experiments, self-identification with the virtual body was modulated predictably by visuo-tactile stimulation. Regarding self-location, we did not find any significant effect within or between experimental conditions or participant groups, although the general pattern was similar. For Experiment 3, we changed the timing of the Mental Ball Dropping task to include responses during the stroking and this may have affected responses. Thus, participants performed the Mental Ball Dropping without knowing exactly when the acoustic cue will be presented, resulting in shorter preparation time for the mental imagery procedure than in Experiments 1 and 2. Secondly, participants performed the Mental Ball Dropping task 6 s earlier as compared to participants of Experiments 1 and 2, allowing less time for the illusion to develop. Finally, we note that the effect size of earlier work and the present Experiments 1 and 2, revealing a modulation of self-location by first-person perspective and visuo-tactile stroking were not very large, making it likely that across several different subjects samples, these effect do not reach significance (averaging data across all three Experiments, we did observe a significant interaction of Group×Stroking).

Regarding the first-person perspective, we confirmed the results of Experiment 2, indicating consistency between online ratings given during the experiment and the final rating of overall first-person perspective-direction. In addition, there was a main effect of Object, with higher frequency of downward direction of the first-person perspective in the body condition than in the no-body, control, condition. This suggests that in the no-body condition participants relied more on vestibular signals, whereas in the body condition (in strong visuo-vestibular conflict), participants were more influenced by the visual information indicating a downward direction of gravity.

## General Discussion

We investigated how multisensory stimulation influences three important aspects of bodily self-consciousness: *self-identification* (i.e. how much ‘I’ identify with a virtual body), *self-location* (i.e. where ‘I’ am located), and *first-person perspective* (i.e. from where ‘I’ perceive the environment). We found three main results. First, self-identification does not depend on the experienced direction of the first-person perspective, whereas self-location does. Second, bodily self-consciousness strongly depends on visual gravitational signals. Third, individual differences in the experienced direction of first-person perspective correlate with individual differences in visuo-vestibular integration, i.e. with idiosyncratic sensory strategies.

### First-person Perspective, Viewpoint, and Self-identification

Results of the present three experiments confirm that self-identification with a virtual body depends on visuo-tactile stimulation and increases during synchronous stroking as observed by previous authors [1], [4], [9], [12]. Whereas in these previous full-body illusion studies the first-person perspective was not altered, we here show that changes in the experienced direction of the first-person perspective did not modulate the strength of self-identification, We thus confirm the data of Ionta et al. [21] in a different subjects sample, using a different stroking robot, in three experiments performed outside the MRI scanner. Whereas our data and those of Ionta et al. [21] suggest that illusory self-identification does not depend on the experienced *direction* of the first-person perspective, previous studies showed that self-identification depends on the *viewpoint* from where the environment is presented to the participants. Thus, Petkova et al. [16] showed that a first-person (i.e. body-centered) viewpoint, but not a third-person viewpoint (i.e. displaced 75 cm to the side), induces stronger illusory self-identification with an artificial body. The data by Petkova and Ehrsson [4] and Slater et al. [17] are also compatible with this observation. These data converge in showing that perceptual changes in the visual direction of the viewpoint modulate self-identification, whereas subjective changes in the direction of the first-person perspective do not, a finding compatible with different brain mechanisms for viewpoint versus first-person perspective changes.

Next, we found that strong visuo-vestibular mismatch diminishes experimentally-induced changes in self-identification. Thus, in Experiment 1, we found that a strong visuo-vestibular conflict (i.e. when observing a body lying on the stomach and seen from an elevated viewpoint) decreases illusory changes in self-identification compared to that obtained with a weak visuo-vestibular conflict (i.e. when observing a body in a standing upright posture seen from a standing viewpoint). These data demonstrate for the first time that visuo-vestibular conflict influences self-identification. They also suggest that the central nervous system extracts visual information about the gravitational influence on body structure and shape, such as gravitational pull on hair, clothes, and shoulder, and modulations in light distribution on the body, suggesting a postural configuration relative to natural light sources. This visual information modulates accordingly the way we identify with fake or virtual bodies seen under the present experimental conditions. Indeed, there is substantial evidence that the visual system is highly tuned to interpret postural configurations in relation with gravity [40] and that the orientation of seen bodies with respect to the apparent direction gravity strongly influences body configuration and body motion processing [46], [47], [48]. This evidence suggests that the central nervous system has internalized the expected influence of gravity on body configuration and structure [40], [49], most likely through mostly preconscious internal models of gravity [50]. Several studies have demonstrated that the vestibular nuclei and the vestibular cortex (such as the temporo-parietal junction) can detect the congruence of visual orientation and the motion of objects with respect to the physical laws of gravity [51], [52], [53]. Thus, in the case of strong visual-vestibular conflict used in the present experiments, these neural systems could detect that the apparent gravitational force acting on the virtual body is incongruent with the physical forces acting on the participant’s body. Such visuo-vestibular conflict may decrease self-identification through a different cortical system than that involved in the visuo-tactile conflicts classically tested, but may involve the temporo-parietal cortex (i.e. [21]). In addition, we note that several related studies demonstrated that pictorial cues about gravitational orientation in a visual scene (which can be artificially tilted or reversed) outweigh orientation information from the physical gravity and the participant’s body [54]. This suggests that visual information about the orientation and direction of gravity strongly constraints the participants’ perception of their own body and the environment.

### Common Multisensory Mechanisms Underlying Self-location and the Experienced Direction of the First-person Perspective

Another main finding of the present results is the close association between self-location and the direction of first-person perspective. Self-location depended both on the synchrony of visuo-tactile stroking and on individual differences in the experienced direction of the first-person perspective. In two out of three experiments, we found that the drift changes in self-location were congruent with the experienced direction of first-person perspective, although visuo-tactile and visuo-vestibular stimulation parameters were identical. These data suggest that these two spatial aspects of bodily self-consciousness are associated in terms of function, multisensory, and likely brain mechanisms [11]. Such a close association between self-location and first-person perspective has also been reported during paroxysmal full-body illusions of neurological origin, such as out-of-body experiences, when the abnormal experience of being located out-of-the body is tightly associated with the experience of perceiving the environment from a disembodied and elevated self-location and perspective [55], [56], [57], [58]. As the commonality between both spatial aspects has been discussed extensively in a recent review, we will not discuss it further here [11].

Another important finding of the present experiments is the influence of visual gravitational signals on the experienced direction of first-person perspective and self-location. We note that, to date, almost all previous behavioral and neuroimaging studies on bodily self-consciousness have used conflicts between visual, tactile, proprioceptive, and motor signals [2], [9], [12], [15], [59], [60]. Therefore, most previous studies neglected to study the contributions of a major sensory system for one’s experience of spatial location and self-motion perception: the vestibular system [58]. In the present study, we did not manipulate vestibular signals directly, but the visual information about the direction of gravity was manipulated, to be congruent or not with the experienced direction of gravity coded by the vestibular sensors. The significant changes in self-location and in the experienced direction of the first-person perspective that we observed in a situation of strong visual-vestibular conflict demonstrated the importance of visual gravitational information for both spatial aspects of bodily self-consciousness. These data are in agreement with previous studies showing that immersion of participants in tilted or inverted visual environments strongly influences the perceived directions of up and down and the perception of the vertical [61], [62]. The vestibular and multisensory nature of the first-person perspective is compatible with data from neurology, vestibular physiology, and abnormal own body perceptions [11], [55], [56], [58]. First, several authors have noted that abnormal forms of the first-person perspective and self-location (such as in out-of-body experiences), occurring in neurological patients and healthy subjects, depend on body position and are more frequent in subjects that are lying supine and still than in subjects sitting or standing upright [63], [64]. This could be related to the decreased sensitivity of otolithic vestibular receptors in the lying position, together with the decrease in motor and somatosensory signals in this position, which could relatively enhance the importance of visual graviceptive signals [65]. Second, observations performed in environments where gravity is strongly reduced (microgravity) or temporarily cancelled (parabolic flights) are associated with strong alterations of self-location. In these conditions, astronauts have reported striking illusions such as body-inversion illusions and room-tilt illusions (e.g. [66]). Likely, such gravitational vestibular manipulations may alter sensory integration in multimodal brain regions [67], decreasing the impact of vestibular, and increasing the importance of visual, tactile and proprioceptive signals. Finally, vestibular brain regions are mostly located at the posterior end of the Sylvian fissure, in close proximity to the temporo-parietal junction, inferior parietal lobule and the intraparietal sulcus [68], [69]. Interestingly, these vestibular regions overlap with the temporo-parietal junction, whose activity has been showed to reflect the experimentally-induced changes in self-location and the experienced direction of the first-person perspective in the full-body illusion [21]. Altogether, these observations indicate that visual and vestibular signals and their integration play a crucial role in the experience of self-location and the subjectively experienced first-person perspective [10].

### Visuo-vestibular Integration and the Experienced Direction of the First-person Perspective

The third main finding of the present experiments regarding the multisensory mechanisms of bodily self-consciousness is that visual-field dependence (measured by the rod and frame test) correlates with the experienced direction of the first-person perspective during the full-body illusion. We found that significantly more visual FD participants experienced a downward (or front) direction of first-person perspective (Down-group) during the full-body illusion while lying supine and being presented with a strong visuo-vestibular conflict. This result suggests an association between a visually dominant style (more deviations of the subjective visual vertical in the rod and frame test) and the subjectively experienced first-person perspective of our participants during the full-body illusion. FD participants rely mostly on an allocentric frame of reference [36], [45] and have been shown to be more unstable than FI participants [22], [23]. We found that the experienced downward direction of the first-person perspective is a relatively unstable perspective. Down-group participants, who experienced mostly a downward direction of the first-person perspective, showed fluctuations of their judgments and only rarely reported to experience a constant downward direction of first-person perspective. This was different for the up-looking participants, who had more stable first-person perspective judgments. Accordingly, it was proposed that FD participants use visual references not only for visual vertical perception, but also to determine their full-body orientation and regulate their balance [23]. Here, we showed that in the full-body illusion FD participants relied more strongly on the gravitational information depicted in the body posture (indicating a dorsal to ventral gravitational acceleration) and adapted the direction of their first-person perspective accordingly (i.e. down-looking). Thus, in this subpopulation, we showed that vision seems to trump vestibular perception for the spatial aspects of bodily self-consciousness, extending previously utilized perceptual paradigms (e.g. [42], [70]) to the first-person perspective. Conversely, we found that more visual FI participants experienced an upward direction of first-person perspective (Up-group) during the full-body illusion under the same experimental conditions. These participants were thus less influenced by visual graviceptive cues and experienced (accurately) that their body was in a supine position and looking upward. FI participants are generally weakly influenced by visual references and have a better balance [23], [36]. It is assumed that they rely mainly on an egocentric (i.e. body-centered) frame of reference and thus presumably rely more strongly on vestibular and somatosensory signals. Indeed, manipulations of proprioceptive signals by head tilts induce stronger deviations of the subjective visual vertical in FI participants [38].

Visuo-vestibular perceptual styles such as visual FD and FI have been described so far during simple visual tasks such as perception of line orientation [71]. The present data suggest that visuo-vestibular styles are also of importance for bodily self-consciousness. Previous studies established connections between visual field dependence-independence and postural control [23], indicating that the reliance upon visual signals constrains one’s body orientation and stabilization. Each individual can refer his body orientation and stabilization to several references frames and this referral depends on a continuous selection along life and environmental constraints [45], [72], [73]. However, no previous studies to date had investigated the contribution of visual field dependence-independence and perceptual styles to higher-level phenomena such as the experienced direction of the first-person perspective. Our data are important because they reveal that the interpretation of the experienced direction of first-person perspective that humans experience continuously and that is a cornerstone of consciousness studies (i.e. [10], [74], [75] depends on sensory strategies, or perceptual styles [45]. The neurobiological understanding of such strategies may allow important insights into the neural mechanisms of self-consciousness.
